# A Lifestyle Intervention to Delay Early Chronic Kidney Disease in African Americans With Diabetic Kidney Disease: Pre-Post Pilot Study

**DOI:** 10.2196/34029

**Published:** 2022-03-15

**Authors:** Mukoso N Ozieh, Leonard E Egede

**Affiliations:** 1 Department of Medicine Division of Nephrology Medical College of Wisconsin Milwaukee, WI United States

**Keywords:** type 2 diabetes mellitus, chronic kidney insufficiency, healthy lifestyle, outcomes research, African Americans, quasiexperimental study

## Abstract

**Background:**

Behavioral factors, such as lifestyle, have been shown to explain approximately 24% of the excess risk of chronic kidney disease (CKD) among African Americans. However, there are limited intervention studies culturally tailored to African Americans with type 2 diabetes mellitus and CKD.

**Objective:**

The main objective of this study was to examine the feasibility and preliminary efficacy of a culturally tailored lifestyle intervention among African Americans with type 2 diabetes mellitus and CKD.

**Methods:**

A pre-post design was used to test the feasibility of a lifestyle intervention in 30 African American adults recruited from the Medical University of South Carolina between January 2017 and February 2017. A research nurse delivered the manualized study intervention weekly for 6 weeks. Clinical outcomes (hemoglobin A_1c_, blood pressure, and estimated glomerular filtration rate [eGFR]) were measured at baseline and postintervention. Disease knowledge, self-care, and behavior outcomes were also measured using validated structured questionnaires at baseline and postintervention. Descriptive statistics and effect sizes were calculated to determine clinically important changes from baseline.

**Results:**

Significant pre-post mean differences and decreases were observed for hemoglobin A_1c_ (mean 0.75%, 95% CI 0.16-1.34; *P*=.01), total cholesterol (mean 16.38 mg/dL, 95% CI 5.82-26.94; *P*=.004), low-density lipoprotein (mean 13.73 mg/dL, 95% CI 3.91-23.54; *P*=.008), and eGFR (mean 6.73 mL/min/1.73m^2^, 95% CI 0.97-12.48; *P*=.02). Significant pre-post mean differences and increases were observed for CKD self-efficacy (mean −11.15, 95% CI −21.55 to −0.75; *P*=.03), CKD knowledge (mean −2.62, 95% CI −3.98 to −1.25; *P*<.001), exercise behavior (mean −1.21, 95% CI −1.96 to −0.46; *P*=.003), and blood sugar testing (mean −2.15, 95% CI −3.47 to −0.83; *P*=.003).

**Conclusions:**

This study provides preliminary data for a large-scale appropriately powered randomized controlled trial to examine a culturally tailored lifestyle intervention in African Americans with type 2 diabetes mellitus and CKD in order to improve clinical, knowledge, self-care, and behavior outcomes in this population.

## Introduction

Chronic kidney disease (CKD), categorized as stage 1 to 5 based on the estimated glomerular filtration rate (eGFR), is a major complication of diabetes and is commonly referred to as diabetic kidney disease (DKD) [1]. DKD is marked by persistent presence of albuminuria (albumin excretion rate ≥30 mg/24 hours; urine albumin to creatinine ratio [UACR] ≥30 mg/g) or a decreased eGFR (<60 mL/min/1.73m^2^). Approximately 31% of individuals with diabetes have DKD [2]. DKD is associated with significant morbidity, cost, a 4- to 5-fold risk of end-stage renal disease, and an increased risk of death [3-6]. African Americans are disproportionately affected by diabetes and DKD compared with non-Hispanic Whites [7]. Further, African Americans are 3 to 4 times more likely to have end-stage renal disease compared with non-Hispanic Whites [4]. The reason for the accelerated progression of kidney disease among African Americans is not completely understood. However, behavioral factors, such as lifestyle, have been shown to explain approximately 24% of the excess risk of CKD among African Americans [8].

Lifestyle modification comprising one or more aspects, including physical activity, diet change, smoking cessation, exercise, skills training, counseling, and stress management, is an essential component of diabetes and CKD management [9-11]. Evidence of the impact of lifestyle interventions on DKD outcomes is limited and conflicting [12,13]. The largest prospective study until date, the Look AHEAD (Action for Health in Diabetes) study, randomly assigned overweight or obese patients with type 2 diabetes to an intensive lifestyle intervention to achieve weight loss compared with a diabetes support and education condition [14,15]. While the intervention was not effective in reducing cardiovascular events (primary outcome), a post-hoc analysis of this study showed that the intensive lifestyle intervention reduced the incidence of very high–risk CKD [15].

The majority of lifestyle intervention studies in patients with DKD are limited by their study design, limited generalizability, small sample size, low proportion of African Americans, or lack of appropriate predefined renal endpoints [12,13,15,16]. In addition, there is a lack of interventions culturally tailored to African Americans, even though evidence suggests that African Americans have a limited understanding of CKD and CKD risk factors [17,18], and lag behind in hemoglobin A_1c_ (HbA_1c_) control, blood pressure control, and use of statins and glucose-lowering medications [19]. Hence, the main objective of this pilot study was to examine the feasibility and preliminary efficacy of a culturally tailored DKD-focused lifestyle intervention on (1) clinical outcomes, (2) disease knowledge, and (3) self-care and behavior outcomes among African Americans with type 2 diabetes and CKD. The study hypothesized that individuals who receive the study intervention will have improved clinical outcomes, disease knowledge, and self-care and behavior outcomes after the intervention.

## Methods

### Ethics Board Review

This study was approved by the Institutional Review Board of the Medical University of South Carolina (Pro#00051414; Institutional Review Board approval date: November 9, 2016).

### Study Design

A pre-post design was used to test the feasibility of a lifestyle intervention with baseline (preintervention) and 2-month (postintervention) assessments. The study participants were non-Hispanic Blacks with type 2 diabetes and CKD, with an eGFR >59 mL/min/1.73m^2^ and a spot UACR of 30-300 mg/g.

### Participants and Setting

Participants were recruited from clinics affiliated with the Medical University of South Carolina between January 2017 and February 2017. Non-Hispanic Black participants were identified and recruited using clinic billing records for ICD-10 (International Classification of Diseases, 10th revision) codes consistent with the diagnosis of type 2 diabetes and cystatin C eGFR >59 mL/min/1.73m^2^, and through referral from physicians and clinic staff. Institutional Review Board–approved study flyers were posted in the clinics, and letters of invitation signed by the clinic director were mailed to patients.

### Screening for Eligibility and Enrollment

Individuals who were aged 21 years or older, self-identified as African American, had a clinical diagnosis of type 2 diabetes and early CKD (stage 1 and 2), were able to communicate in English, and had a telephone (landline or cell phone) were eligible to participate in the study. Individuals who had cognitive impairment, alcohol or drug abuse, acute decompensation of chronic disease conditions, CKD of stage 3 or higher, malignancy, life-expectancy of less than 6 months, and other known disease conditions causing proteinuria were excluded from the study. Transplant recipients, individuals participating in another diabetes or CKD trial, and those who did not have telephone access were not eligible for the study.

A total of 30 participants who met the inclusion criteria were enrolled by a research assistant. Eligible participants received up to US $150 in compensation for completing all the study assessments (screening, baseline, and 2-month study assessment).

### Description of the Intervention

The study intervention was adapted from a culturally tailored study, Technology-Intensified Diabetes Education and Skills Training Intervention (TIDES) [20], and was tailored to focus on DKD. The study intervention was based on the Information-Motivation-Behavioral Skills model and provides information, motivation, and behavioral skills training (using motivational enhancement techniques) [21]. Patients were assigned the FORA 2-in-1 Telehealth System at the beginning of the study and provided glucose test strips to allow testing at least once a day.

All intervention sessions were telephone-delivered weekly by a research nurse for 6 weeks. Intervention sessions lasted 30 minutes, and the research nurse was trained in behavioral skills counseling and the study intervention content. The study educational materials were developed based on the National Kidney Disease Education Program [22] and written in lay language for African Americans with DKD. The description of weekly content is provided in Multimedia Appendix 1. All education sessions were audiotaped, and 20% were randomly selected and reviewed by the principal investigator to ensure the research nurse delivered the intervention appropriately. All study participants received behavioral skills training focused on 3 lifestyle behaviors (physical activity, diet, and medication adherence). Target lifestyle behavior goals were set in collaboration with the patients and were guided by current problem areas and preferences.

### Study Measures and Data Collection Schedule

Participant information was collected using validated questionnaires administered at 2 time points: at baseline and 2 months postintervention (see Multimedia Appendix 2). Study data were obtained by a trained research assistant.

### Feasibility Measures

Feasibility measures were recruitment, session attendance rate, and dropout proportion.

### Outcome Measures

BMI was calculated using weight in kg and height in m^2^. Blood pressure readings were obtained using automated blood pressure monitors at baseline and 2 months. The device was programmed to take 3 readings at 2-minute intervals, and the readings were averaged. UACR was measured at baseline and 2-month visits using spot urine. Blood samples were assayed for HbA_1c_, cholesterol, and eGFR at baseline and 2 months by a trained nurse. The Patient Health Questionnaire-9 (PHQ-9), a brief questionnaire that scores each of the 9 DSM-IV (Diagnostic and Statistical Manual of Mental Disorders, fourth edition) criteria, was used to assess for depression [23]. See Multimedia Appendix 2 for details on outcome and process measures.

### Process and Behavioral Measures

#### CKD Self-efficacy Scale

This involved a 25-item instrument that measures disease-related self-efficacy in the following 4 core areas: (1) autonomy, (2) self-regulation, (3) problem solving, and (4) seeking social support [24]. The Cronbach alpha coefficient for the total scale was .94, and the value for each of the 4 subscales ranged from .84 to .90 [24].

#### CKD Knowledge Questionnaire

This involved a 28-item Kidney Knowledge survey, which has good internal consistency and high reliability (coefficient of 0.72) [25].

#### Diabetes Knowledge Questionnaire

This involved the 24-item Diabetes Knowledge Questionnaire, which has a reliability coefficient of 0.78 [26].

#### Health Literacy

This was measured using the 3-item Chew health literacy scale, which assesses the capacity to obtain, process, and understand basic health-related decisions [27]. The 3 questions have been shown to be effective in detecting inadequate health literacy (areas under the receiver operating characteristic curve of 0.87, 0.80, and 0.76, respectively) [27].

#### Behavioral Skills

This was assessed with the Summary of Diabetes Self-Care Activities (SDSCA) scale [28]. It is a brief, validated, self-report questionnaire of diabetes self-management that includes items assessing diet, exercise, medication adherence, and self-blood glucose testing. The average interitem correlations within scales were high, test-retest correlations were moderate, and correlations with other measures of diet and exercise generally supported the validity of the subscales.

### Statistical Analyses

Important measures of feasibility analysis included recruitment, session attendance rate, and dropout proportion. We used 95% CIs for proportions to estimate (1) the proportion of participants who agreed to participate among those who were initially approached, (2) the proportion who were compliant with the treatment intervention, and (3) the proportion who dropped out. In addition, frequency distributions describing the participants’ reasons for noncompliance and discontinuation of study participation will be provided.

For quantitative analysis, univariate descriptive statistics and frequency distributions were calculated for the total sample. Pre-post mean differences were tested using paired *t* tests. Effect size, a measure of treatment effect, was used to interpret the effects of the intervention. An effect size of 0.2 was considered small, 0.5 was considered moderate, and 0.8 was considered large. In addition to effect sizes, which demonstrated clinical relevance, a statistically significant difference was noted for the primary measurement. All statistical analyses were performed using Stata software (StataCorp).

## Results

### Study Profile

**Figure 1:**
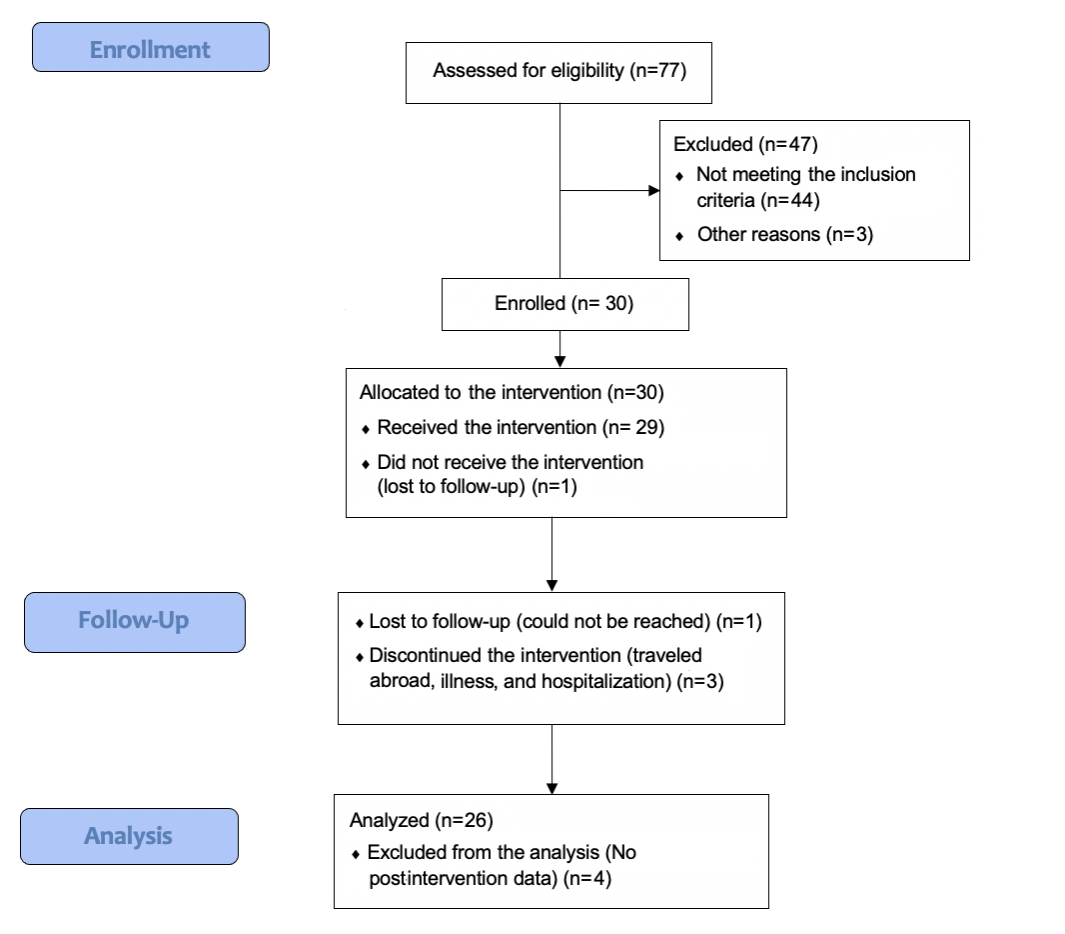
Study flow diagram.

Between January 9, 2017, and April 28, 2017, 77 patients were screened, and 30 eligible patients were enrolled into the study (Figure 1). All 30 (100%) patients completed the baseline assessment, and 26 (87%) completed assessments at 2 months. Four (13%) participants were lost to follow-up; hence, the analytical sample included 26 participants.

### Baseline Demographic Profile

Table 1 shows the baseline characteristics of the study participants. The mean age of the study participants was 57 years, and the mean duration of diabetes was 14 years. The majority were female (21/30, 70%), unmarried (17/30, 57%), unemployed (20/30, 67%), and insured (30/30, 100%). Over half of the participants reported having a “good” health status (16/30, 53%) and not using any special equipment (16/30, 53%).

**Table 1:** Baseline sample demographic characteristics of African Americans with type 2 diabetes and chronic kidney disease enrolled in the pre-post study.

Variable		Value (N=30)
Age (years), mean (SD)		56.7 (13.5)
**Sex, n (%)**		
	Female	21 (70)
**Marital status, n (%)**		
	Married	13 (43)
**Employment status, n (%)**		
	Employed	10 (33)
**Income, n (%)**		
	<US $25,000	18 (60)
**Smoking status, n (%)**		
	Smoker	9 (30)
**Insurance status, n (%)**		
	Insured	30 (100)
**Health status, n (%)**		
	Fair/poor	14 (47)
**Use of special equipment, n (%)**		
	Yes	14 (47)
**Physical activity days per week, n (%)**		
	0	16 (53)
	1+	14 (47)
Years of education, mean (SD)		12.6 (1.7)
Work hours per week, mean (SD)		14.3 (24.0)
Duration of diabetes (years), mean (SD)		14.2 (9.1)

### Feasibility Findings

Overall, 33 out of 77 (43%) participants contacted were eligible for the study, and 30 participants were successfully recruited for this study. Among the 30 participants, 21 (70%) completed all 6 sessions, 26 (87%) completed assessments at 2 months, and 1 (3%) dropped out of the study. The reasons for incomplete sessions were death in the family, illness, or hospitalization. One participant dropped out after enrollment because of hospitalization for pneumonia.

### Preintervention and Postintervention Differences in Clinical Outcomes

Table 2 presents preintervention (baseline) and postintervention (2 months) differences in clinical outcomes. Significant preintervention and postintervention mean differences and decreases were observed for HbA_1c_ (mean 0.75; *P*=.01), total cholesterol (mean 16.38; *P*=.004), low-density lipoprotein (LDL) (mean 13.73; *P*=.008), and eGFR (mean 6.73; *P*=.02). We observed nonstatistically significant increases in BMI (mean −0.48; *P*=.05), systolic blood pressure (mean −1.77; *P*=.61), diastolic blood pressure (mean −3.42; *P*=.21), and the UACR (mean −18.63; *P*=.79). There were also nonstatistically significant decreases in the PHQ-9 score for depression (mean 1.30; *P*=.17), high-density lipoprotein (mean 1.70; *P*=.23), and triglycerides (mean 4.03; *P*=.62).

**Table 2:** Preintervention and postintervention mean differences in the clinical outcomes of African Americans with type 2 diabetes and chronic kidney disease enrolled in the pre-post study.

Variable	Baseline^a^, mean (SD)	2 months^a^, mean (SD)	Mean difference (95% CI)	*P* value
BMI (kg/m^2^)	35.9 (8.1)	36.4 (8.4)	−0.48 (−0.96 to 0.01)	.05
Systolic blood pressure (mmHg)	131.2 (17.9)	133.0 (15.2)	−1.77 (−8.84 to 5.30)	.61
Diastolic blood pressure (mmHg)	77.0 (10.2)	80.5 (9.5)	−3.42 (−7.80 to 0.96)	.12
Hemoglobin A_1c_ (%)	9.1 (2.5)	8.3 (1.9)	0.75 (0.16 to 1.34)	.01
Total cholesterol (mg/dL)	199.5 (44.9)	183.1 (34.6)	16.38 (5.82 to 26.94)	.004
Low-density lipoprotein (mg/dL)	122.2 (41.4)	108.4 (31.1)	13.73 (3.91 to 23.54)	.008
High-density lipoprotein (mg/dL)	50.4 (11.0)	48.7 (12.8)	1.70 (−1.14 to 4.52)	.23
Triglycerides (mg/dL)	134.3 (58.3)	130 (61.6)	4.03 (−12.80 to 20.88)	.62
Microalbumin-creatinine ratio	264.8 (375.5)	283.5 (557.6)	−18.63 (−168.83 to 131.58)	.79
Glomerular filtration rate (mL/min/1.73m^2^)	93.4 (21.9)	86.7 (21.7)	6.73 (0.97 to 12.48)	.02
Patient Health Questionnaire-9 (PHQ-9) score	5.2 (4.9)	3.9 (4.5)	1.30 (−0.62 to 3.23)	.17

^a^Baseline is preintervention and 2 months is postintervention.

### Preintervention and Postintervention Differences in Knowledge, Self-care, and Behavior Outcomes

Table 3 displays participant responses to questions related to knowledge, skills, self-care, and behavior outcomes. Significant preintervention and postintervention mean differences and increases were observed for CKD self-efficacy (mean −11.15; *P*=.03), CKD knowledge (mean −2.62; *P*<.001), exercise behavior (mean −1.21, *P*=.003), and blood sugar testing (mean −2.15; *P*=.003). We observed nonstatistically significant increases in diet (mean −0.42; *P*=.49), special diet (mean −0.43; *P*=.23), and foot care (mean −0.21; *P*=.60). A nonstatistically significant decrease in diabetes knowledge (mean 0.77; *P*=.25) was also observed, while no significant change in health literacy (mean 0.04; *P*=.66) was seen.

**Table 3:** Pre-post mean differences in knowledge, self-care, and behavior outcomes of African Americans with type 2 diabetes and chronic kidney disease enrolled in the pre-post study.

Variable		Baseline^a^, mean (SD)	2 months^a^, mean (SD)	Mean difference (95% CI)	*P* value
**Knowledge**					
	CKD^b^ self-efficacy	208.9 (36.0)	220.0 (27.4)	−11.15 (−21.55 to −0.75)	.03
	DM^c^ knowledge	16.2 (3.8)	15.4 (3.7)	0.77 (−0.59 to 2.14)	.25
	CKD knowledge	18.4 (3.8)	21.0 (2.1)	−2.62 (−3.98 to −1.25)	<.001
	Health literacy	0.3 (0.5)	0.3 (0.5)	0.04 (−0.14 to −0.21)	.66
**Self-care and behavior**					
	Diet	3.9 (2.1)	4.4 (2.1)	−0.42 (−1.68 to 0.83)	.49
	Special diet	4.0 (1.4)	4.4 (1.2)	−0.43 (−1.16 to 0.30)	.23
	Exercise behavior	1.8 (1.7)	3.0 (1.6)	−1.21 (−1.96 to −0.46)	.003
	Blood sugar test	3.7 (2.6)	5.9 (1.8)	−2.15 (−3.47 to −0.84)	.003
	Foot care	4.9 (2.2)	5.1 (2.1)	−0.21 (−1.05 to 0.63)	.60

^a^Baseline is preintervention and 2 months is postintervention.

^b^CKD: chronic kidney disease.

^c^DM: type 2 diabetes.

## Discussion

### Principal Findings

This study examined the feasibility and preliminary efficacy of a culturally tailored DKD-focused lifestyle intervention in African Americans with type 2 diabetes and CKD. With 100% recruitment, a 70% session attendance rate, and a 3% drop-out rate, the study findings suggest that the design, recruitment, and delivery of a culturally tailored lifestyle intervention for high-risk African Americans with type 2 diabetes and CKD are feasible. This study was also designed to examine preliminary changes in clinical outcomes, disease knowledge, self-care, and behavior outcomes. We observed statistically significant changes in the clinical outcomes of HbA_1c_, total cholesterol, LDL, and eGFR following the study intervention. In addition, there were statistically significant increases in CKD self-efficacy, CKD knowledge, exercise. and blood sugar testing.

### Comparison With Prior Work

Behavior lifestyle intervention trials have conflicting results on the impact of lifestyle interventions on clinical outcomes [12]. Consistent with our study findings, a systematic review by Van Huffel et al evaluating the impact of exercise and diet on health outcomes in individuals with diabetes and CKD concluded that exercise and diet interventions have beneficial effects on glycemic control, BMI, and quality of life [13]. Similarly, large trials, such as the “Reduction of Endpoints in NIDDM with the Angiotensin II Antagonist Losartan (RENAAL),” “Action to Control Cardiovascular Risk in Diabetes (ACCORD),” “Antihypertensive and Lipid-Lowering Treatment to Prevent Heart Attack Trial (ALLHAT),” and “Action in Diabetes and Vascular Disease: Preterax and Diamicron Modified Release Controlled Evaluation (ADVANCE),” demonstrated that aggressive risk factor control in African Americans using antihypertensive, antihyperglycemic, or lipid-lowering medications is beneficial [29-33]. However, these studies focused on risk factor control using medications and did not emphasize lifestyle modification, which is a core component of diabetes and CKD management [3,9,10].

Contrary to our study findings, a systematic review and meta-analysis of self-management support interventions for people with diabetes and CKD showed that these interventions may improve self-care activities, HbA_1c_, and systolic blood pressure [34]. While we observed a significant increase in CKD self-efficacy, disease knowledge, exercise behavior, and blood sugar testing, our study did not show a significant difference in blood pressure; however, it was not powered to confirm or refute a hypothesis, which could explain the lack of statistical significance in most clinical outcomes. The impacts of lifestyle interventions on kidney function are also inconsistent, with some studies demonstrating no effect, or a negative or positive effect [12,13]. We observed a significant negative effect (decrease) in the eGFR postintervention in our study population. Glomerular hyperfiltration often mediated by hyperglycemia results in a high eGFR in type 2 diabetes and is a hallmark finding in DKD [35]. Nonpharmacological interventions, such as decreases in body weight, and salt and protein intake, have been shown to ameliorate diabetic hyperfiltration [35]. It is unclear why we observed these findings given the lack of a significant difference in BMI or dietary habits. Future large-scale and more rigorous behavior lifestyle randomized controlled trials in this population should explore measuring changes in salt and protein intake, and examine the impact on outcomes.

Recent evidence demonstrates that glucose-lowering medications, such as glucagon-like peptide 1 receptor agonists and sodium-glucose cotransporter-2 inhibitors, are of particular benefit in the prevention and treatment of CKD in patients with type 2 diabetes [36]. However, despite the strengths of these large clinical trials [37-44] and limited data on the efficacy of lifestyle interventions in African Americans [12,15,34], African Americans remain poorly represented. It is established that African Americans with CKD are poorly represented in clinical trials [45], and low inclusion of African Americans in clinical trials limits the generalizability of study findings. This potentially propagates existing disparities in a high-risk high-cost population. Low participation of African Americans in clinical trials is often attributed to poverty, lack of accessibility, lack of information on clinical trials, and chronic disease–related stigma [45,46]. There is a need to overcome these barriers and increase the participation of African Americans in clinical trials. Ongoing clinical trials are exploring novel community-based screening recruitment methods for African Americans with CKD [46,47]. More intervention studies that focus on high-risk patients incorporating such novel recruitment strategies are needed. In addition, behavioral lifestyle interventions that account for contextual factors facing high-risk African American populations with diabetes and CKD are needed [48].

### Strengths, Limitations, and Future Direction

The findings of this study are promising and have important clinical implications. Significant changes observed in clinical outcomes, such as a decrease in HbA_1c_, and improved CKD knowledge, self-care, and behavior, can prevent or delay the progression of CKD to renal failure, and improve quality of life and survival in this study population. This could potentially reduce the economic burden associated with renal failure and the life-threatening complications of renal failure. Despite these promising findings, some limitations are worth noting. First, the relatively small sample size, limited intervention duration, and lack of a control group might have affected the findings. However, the goal of this feasibility pilot study was to generate information needed for planning and designing a future large-scale study. Second, eGFR was estimated using creatinine and cystatin C equations with race. Recent evidence suggests that the inclusion of race in eGFR estimation overestimates measured eGFR, which potentially exacerbates health disparities and contributes to systemic racism. While it is unlikely that the eGFR equation used for this study influenced the study findings, future studies will use new creatinine and cystatin C equations without race to ensure accuracy. Third, although majority of the study participants completed all intervention sessions, some weekly intervention sessions were delayed. The main reasons for delayed intervention sessions were travel abroad, hospitalization, and death in the family. Future studies will incorporate a run-in period to establish expectations and processes for timely completion of intervention sessions in the event of hospitalization or unanticipated events. In addition, we will account for loss of information due to dropout when calculating sample size. Fourth, the study findings may not be generalizable to other populations since the study was primarily designed for African American/non-Hispanic Black populations.

### Conclusion

This study clarifies the feasibility and preliminary efficacy of a culturally tailored DKD-focused lifestyle intervention in African Americans with type 2 diabetes and CKD in terms of clinical, knowledge, self-care, and behavior outcomes. Statistically significant changes in the clinical outcomes of HbA_1c_, total cholesterol, LDL, and eGFR were observed following the study intervention. In addition, there were statistically significant increases in CKD self-efficacy, CKD knowledge, exercise, and blood sugar testing. Based on the results of this study, a trial to determine the efficacy of this intervention would be feasible in African Americans with type 2 diabetes and CKD. The findings from this study will also serve as preliminary data to inform the design of a large-scale appropriately powered randomized controlled trial to examine the efficacy of a culturally tailored lifestyle intervention in African Americans with comorbid diabetes and CKD in terms of clinical, knowledge, self-care, and behavior outcomes.

## Appendix

Multimedia Appendix 1 Pre-post study intervention sessions and intervention content.

Multimedia Appendix 2 Data collection schedule and measures for the pre-post study intervention.

## Abbreviations

CKD: chronic kidney disease
DKD: diabetic kidney disease
eGFR: estimated glomerular filtration rate
HbA_1c_: hemoglobin A_1c_
LDL: low-density lipoprotein
PHQ-9: Patient Health Questionnaire-9
SDSCA: Summary of Diabetes Self-Care Activities
UACR: urine albumin to creatinine ratio
