# Supplementary material for: A Lifestyle Intervention to Delay Early Chronic Kidney Disease in African Americans With Diabetic Kidney Disease: Pre-Post Pilot Study
Source: JMIR Form Res. 2022 Mar 15;6(3):e34029. doi: 10.2196/34029 (PMC8965678; doi:10.2196/34029)
Supplement: Multimedia Appendix 1 [file formative_v6i3e34029_app1.docx]

**Multimedia Appendix 1.** Pre-post study intervention sessions and intervention content.

| **Sessions** | **Content** |
| --- | --- |
| **1** | - Meet RN face-to-face, study details, contact information,  receive FORA device, test strips & device set up  - Diabetes and Chronic Kidney Disease (CKD) overview, self-monitoring using the devices |
| **2** | - CKD Basics: Causes, Stages and Diagnosis of CKD  - RN reviews weekly FORA readings; skills training on 1 of 3 lifestyle behaviors (physical activity, diet, medication adherence) |
| **3** | - Slowing progression of CKD  - RN reviews FORA readings & skills training |
| **4** | - Relevance of medication adherence in CKD  - RN reviews FORA readings & skills training |
| **5** | - Diet and CKD  - RN reviews FORA readings; skills training |
| **6** | - Exercise and Smoking in CKD  - RN reviews FORA readings & skills training |
