# Supplementary material for: A Lifestyle Intervention to Delay Early Chronic Kidney Disease in African Americans With Diabetic Kidney Disease: Pre-Post Pilot Study
Source: JMIR Form Res. 2022 Mar 15;6(3):e34029. doi: 10.2196/34029 (PMC8965678; doi:10.2196/34029)
Supplement: Multimedia Appendix 2 [file formative_v6i3e34029_app2.docx]

**Multimedia Appendix 2.** Data collection schedule and measures for the pre-post study intervention.

| **Questionnaires and measurements** | **Baseline visit** | **2-month visit** |
| --- | --- | --- |
| **Feasibility measures** | | |
| Recruitment |  | X |
| Session attendance |  | X |
| Dropout proportions |  | X |
| **Outcome measures** | | |
| Urine Albumin to Creatinine ratio | X | X |
| Estimated Glomerular Filtration Rate | X | X |
| Blood Pressure | X | X |
| Hemoglobin A1c | X | X |
| Low Density Lipoprotein | X | X |
| **Process measures** | | |
| ^a^CKD knowledge questionnaire | X | X |
| ^b^Diabetes knowledge questionnaire | X | X |
| ^c^Chronic kidney disease self-efficacy scale | X | X |
| ^d^Behavioral skills (Summary of Diabetes Self-Care Activities; SDSCA) | X | X |
| Treatment Credibility |  | X |
| **Covariates** | | |
| Patient demographics | X |  |
| ^e^Chew Health literacy screening | X | X |
| Patient Health Questionnaire | X | X |
| Medical comorbidity (chronic health conditions, BRFSS) | X | X |
| Body Mass Index | X | X |

^a^CKD knowledge questionnaire: The 28-question survey includes 5 multiple choice type questions and 23 Yes-No questions. To assess the survey score, 1 point awarded for each correct answer and zero for each wrong one; none of the questions included an "I don’t know" option. The questionnaire doesn’t have domains, and the total score was calculated as the sum of the correct responses to each question divided by the total number of questions, which results in values from 0 to 1 where 1 means the highest level of knowledge;

^b^Diabetes knowledge questionnaire: The 24-question survey has three response options “yes”, “no”, and “don’t know”. One point is awarded for each correct option and no point or negative scoring for wrong answer. A higher score represents better disease knowledge.

^c^CKD self-efficacy scale: Response scale ranges from “1” (least relevant) to “4” (most relevant); ^d^Behavioral skills (Summary of Diabetes Self-Care Activities; SDSCA): 11 questions, which measure the frequency of performing diabetes self-care activities over the last seven days including diet, exercise, blood glucose testing, foot care and tobacco use. The participant marks the number of days on which the indicated behavior was performed on an eight-point Likert scale to answer the questions. The first ten items are summed to a total score as well as to four sub scores: diet (item 1–4), exercise (item 5–6), blood-glucose testing (item 7–8) and foot-care (item 9–10). The eleventh item focuses on smoking habits and assesses the average number of cigarettes smoked per day. All scale scores range from 0 to 7 with higher scores suggesting better self-management.

^e^Chew Health literacy screening: 3 survey items with Likert-type responses (0-5). Any response that is 3 or greater on any question indicates inadequate health literacy.
